# A feasibility study of educational tools for osteomalacia

**DOI:** 10.1007/s10067-016-3451-2

**Published:** 2016-10-26

**Authors:** R. Waxman, A. Adebajo, S. Robinson, D. Walker, M. Johnson, A. Rahman, A. Samanta, K. Kumar, K. Raza, P. Helliwell

**Affiliations:** 1 Leeds Institute of Rheumatic and Musculoskeletal Medicine, University of Leeds, 2nd Floor, Chapel Allerton Hospital, Harehills Lane, Leeds, LS7 4SA UK; 2 Faculty of Medicine, Dentistry and Health, University of Sheffield, Beech Hill Road, Sheffield, S10 2RX UK; 3 Northumbria NHS Trust, North Tynside General Hospital, Rake Lane, North Sheilds, NE29 8NH UK; 4 Mary Seacole Research Centre, De Monfort University, The Gateway, Leicester, LE1 9BH UK; 5 Department of Rheumatology, University College London, Rayne Institute, 5 University Street, London, WC1E 6JF UK; 6 University Hospitals of Leicester NHS Trust, Leicester, LE1 5 WW UK; 7 Faculty of Biology, Medicine and Health, The University of Manchester, Oxford Road, Manchester, M13 9PL UK; 8 Institute of Inflammation and Ageing, College of Medical and Dental Sciences, University of Birmingham, Edgbaston, Birmingham, B15 2TT UK; 9 Department of Rheumatology, Sandwell and West Birmingham Hospitals NHS Trust, Birmingham, B18 7QH UK

**Keywords:** Community research, Ethnic minorities, Osteomalacia, Patient education

## Abstract

Many people in the UK, particularly people of South Asian origin, are advised to supplement their vitamin D intake, yet most do not. This suggests an unmet educational need. The osteomalacia mind map was developed to meet this need. The mind map contains culturally sensitive images, translated into Urdu and made interactive on a DVD. This study explores the feasibility of a randomised controlled study to measure the effect of education on improving vitamin D knowledge and adherence. This was a pilot and feasibility study. Cluster randomisation was used to avoid inter person contamination. Two South Asian women’s groups were recruited to receive information about osteomalacia either by interactive DVD or an Arthritis Research UK leaflet. Knowledge and compliance were tested before and after the educational interventions via a knowledge questionnaire and the measurement of vitamin D and parathormone levels. The groups were found to be mismatched for knowledge, educational attainment and language at baseline. There were also organisational difficulties and possible confounding due to different tutors and translators. The DVD group had high knowledge at baseline which did not improve. The leaflet group had low knowledge at baseline that did improve. The DVD group had lower parathormone which did not change. The leaflet group had an increase in vitamin D but parathormone remained high. Performing a randomised study with this population utilising an educational intervention was difficult to execute. If cluster randomisation is used, extreme care must be taken to match the groups at baseline.

## Introduction

Osteomalacia is a generalised bone condition in which there is inadequate mineralization of the bone. The most common cause of osteomalacia in the UK is insufficient vitamin D. Bone pain, tenderness, muscle weakness and difficulty in walking are all common manifestations of osteomalacia.

Osteomalacia and vitamin D deficiency are highly prevalent among some ethnic minority populations in the UK [1, 2]. There are a number of reasons for this: limited sunlight in the UK, dark skin limiting access to vitamin D from weak sunlight, dietary habits, mode of dressing, direct sunlight avoidance and genetic factors. Osteomalacia is associated with significant functional limitation and reduced quality of life, and may contribute to the high incidence of widespread pain among individuals of South Asian background [2–5]. However, simple modifications to lifestyle, dietary habits and inexpensive oral supplementation can significantly reverse the disease [2, 3].

The World Health Organisation defines health education as ‘any combination of learning experiences designed to help individuals and communities improve their health by increasing their knowledge or influencing their attitudes. Ethnic minority participants have significant and distinct health education needs [6–9]. A variety of complex factors contributes to their unique requirements including extent of education and training, socioeconomic status and fluency and confidence in reading English. Educational resources must be culturally appropriate and sensitive if key learning objectives, such as conveying understanding of a disease and informing health decision-making, are to be achieved [7–9]. It is essential that these factors are addressed in the provision of health education for ethnic minority populations [6, 10]. Traditional educational materials, such as printed leaflets, may act as barriers to changing knowledge, belief and behaviour [8]. Research also suggests that community approaches to health education may be especially attractive and relevant for people from ethnic minority groups [11, 12].

Arthritis Research UK (AR-UK) has produced a range of educational resources focussed on osteomalacia to address the needs of people of South Asian background, including a mind map derived from their osteomalacia leaflet. The purpose of a mind map is to make information more accessible to those with limited reading skills and/or those for whom English is not a first language. A mind map is a potentially powerful educational tool particularly when the target audience has been involved in its development [13]. There are various definitions of a mind map. In this document, we are referring to a diagram that presents information as pictures. Mind maps have been found to be beneficial to British Caucasian populations with rheumatoid arthritis [14]. The elements of the AR-UK osteomalacia mind map have been previously validated [15].

AR-UK has also produced a short version of the osteomalacia booklet translated into Bengali, Gujarati, Hindi, Punjabi and Urdu, and an audio CD on Osteomalacia. These resources have been evaluated using qualitative and quantitative methodologies and have been found to be beneficial [16, 17]. But, there is evidence to suggest that people from ethnic minority groups prefer educational information in formats other than printed, possibly because of relatively low levels of literacy [18, 19]. Electronic educational resources have the potential to convey health education messages more effectively than printed leaflets as information can be presented according to the needs of the individual or group. Electronic resources allow for information to be ‘layered’, with very basic information presented at the ‘top layer’ and subsequent layers covering more in-depth topics. The advantage of this presentation is that each layer can provide a comprehensive coverage (width) of the topic, whilst allowing the individual to choose the depth, or amount of detail, that is suitable for them. This method of delivering health education is consistent with recent AR-UK educational policy.

## The osteomalacia DVD

The osteomalacia DVD is based upon the content of the AR-UK osteomalacia leaflet and the AR-UK osteomalacia mind map. Content has been divided into three sections, or layers, linked to icon images. Each layer offers more detail on the selected topics, with onward links to successively more detailed information. Users may elect to navigate any route through the package, going to whatever depth they wish. Information is presented in English or Urdu at the top layer (the green layer) and English at the deeper levels (layer 2 is red and layer 3 is blue).

The osteomalacia DVD tool was first reviewed by the OMMIT (Osteomalacia Mind Map Interactive Tool) Project Advisory Group, consisting of IT, literacy and ethnicity professionals with an expertise in osteomalacia and education. The final DVD tool was then evaluated by an Urdu-speaking population using both qualitative and quantitative methodologies [15].

The aim of this feasibility educational intervention study was to test this electronic, interactive mind map of osteomalacia against the AR-UK printed leaflet on the subject, in a community-based group education setting, assisted by an Urdu-speaking tutor and delivered to individuals of South Asian background. Our hypothesis was that the delivery of an electronic resource would be more effective at changing health knowledge and behaviour than a printed leaflet. A secondary aim was to explore the association between knowledge about osteomalacia and vitamin D and parathyroid hormone (PTH) levels.

## Methods

Full ethical approval was obtained for this study. Two community-based venues in the Bradford area agreed to approach South Asian women’s groups who met in their centres on our behalf. A cover letter and study information sheet, in both Urdu and English, were distributed by the centres to potential participants.

Two women’s groups were recruited from the two separate South Asian community centres: group 1, (the leaflet group) and group 2 (the DVD group). Both groups attended two educational sessions, 6 weeks apart, between the 28th January and the 15th April 2015. An Urdu translator provided simultaneous translation at all sessions. In the baseline session, both groups gave informed written consent and were asked to complete the Osteomalacia Knowledge Questionnaire (OKQ). The OKQ is an eight-item questionnaire developed and adapted from the Patient Knowledge Questionnaire [20]. Basic demographic information and a 10-ml serum sample were also collected. Basic demographic information included date of birth, ethnicity, spoken language, education and skin exposure to sunlight. Laboratory tests included urea and electrolytes (U&E), calcium, phosphate, alkaline phosphatase (ALP), vitamin D and PTH. Levels of 25-OH vitamin D less than 75 nmol/L were considered deficient. Levels of PTH greater than 7.6 pml/L were considered high. High PTH levels are associated with osteomalacia. Group 1 received a short PowerPoint presentation on vitamin D and Osteomalacia based upon the standard information given routinely to patients at risk of developing osteomalacia attending the rheumatology department at St Luke’s Hospital, Bradford, together with an English and/or Urdu version of the AR-UK leaflet on Osteomalacia to take home. Group 2 received an introductory presentation of the AR-UK Osteomalacia Mind Map DVD and were each given copies of the DVD to take home. Both groups were asked to complete the OKQ and the Medication Adherence Report Scale (MARS) at the follow-up session 6 weeks later [21]. The Medication Adherence Report Scale is a six-item questionnaire developed to test medicine adherence. A 10-ml serum sample was also collected at the second visit. All sessions were conducted in English with two Urdu translators; one provided simultaneous translation of the presentations and both assisted in completing forms and questionnaires which were also in English. Letters were sent to each participant upon completion of the study detailing the results of their laboratory tests.

## Results

Groups were similar in terms of age and primary language (Table 1), but not all were Urdu speakers. The groups were dissimilar in terms of ethnic origin and educational attainment. The DVD group included people from the Middle East and Mauritius, and these individuals were more highly educated than anyone in the leaflet group.

**Table 1:** Demographics

*n* (%)	Group 1 (leaflet)	Group 2 (DVD)
Attended baseline	16	11
Attended follow-up	12 (75 %)	9 (82 %)
Mean age, years (95 % CI) all	51 (44, 57)	51 (39, 61)
Mean age, years (95 % CI) attended both sessions	51 (43, 58)	48 (36, 59)
Ethnicity (all / attended both sessions)		
Pakistani	11 (69 %) / 7 (58 %)	8 (73 %) / 6 (67 %)
Indian	5 (31 %)	0
Other (Iraqi, Egyptian, Mauritian)	0	3 (27 %) / 3 (33 %)
1st Language (all / attended both sessions)		
Urdu	8 (50 %) / 5 (42 %)	6 (55 %) / 4 (44 %)
Punjabi	4 (25 %) / 3 (25 %)	2 (18 %) / 2 (22 %)
Gujurati	3 (19 %) / 3 (25 %)	0
Other	1 (16 %) / 1 (8 %)	3 (27 %) / 3 (34 %)
2nd Language (all / attended both sessions)		
Urdu	3 (27 %) / 3 (30 %)	2 (18 %) / 2 (22 %)
English	8 (73 %) / 7 (70 %)	9 (82 %) / 7 (78 %)
Education (all / attended both sessions)		
None	4 (25 %) / 1 (8 %)	2 (18 %) / 1 (11 %)
Primary	4 (24 %) / 4 (33 %)	4 (36 %) / 3 (33 %)
Secondary	6 (38 %) / 5 (42 %)	2 (18 %) / 2 (22 %)
Higher	2 (13 %) / 2 (17 %)	3 (27 %) / 3 (33 %)
How often do you expose your skin to sun? (all / attended both sessions)		
Never	0	1 (9 %) / 1 (11 %)
Rarely	5 (31 %) / 5 (42 %)	5 (46 %) / 3 (33 %)
Sometimes	2 (19 %) / 1 (8 %)	0
1–2/week	8 (50 %) / 6 (50 %)	3 (27 %) / 3 (33 %)
Every day	0	2 (18 %) / 2 (22 %)

The DVD group scored much higher on the OKQ at baseline (Table 2) but their scores increased less (and in some cases were reversed) at follow-up. This was despite reviewing the DVD, as a group, three times between sessions.

**Table 2:** Knowledge, vitamin D, and parathormone levels at baseline and follow-up

	OKQ Median (IQR)		Vitamin D mean (95 % CI)		Parathyroid mean (95 % CI)	
	Group 1 (leaflet)	Group 2 (DVD)	Group 1 (leaflet)	Group 2 (DVD)	Group 1 (leaflet)	Group 2 (DVD)
Baseline—all	5.50 (2.25,18.00)	18.00 (10.00,23.00)	44.19 (34.56,53.81)	49.00 (34.44,63.56)	8.87 (6.37,11.37)	5.78 (3.82,7.74)
Baseline—attended follow-up	7.50 (2.50,19.50)	18.00 (10.00,25.50)	43.00 (29.94,56.06)	49.00 (31.41,66.59)	9.78 (6.47,13.10)	5.59 (3.03,8.14)
Follow-up	23.00 (15.50,28.50)	20.00 (15.00,24.00)	57.08 (46.43,67.73)	33.22 (14.47,51.97)	10.21 (2.17,15.25)	5.83 (3.53,8.12)

There were no differences in the blood results at baseline or follow-up between groups for creatinine, ALP, calcium, hydroxy vitamin D or PTH level. However, phosphate levels were slightly higher in the leaflet group at baseline. Parathyroid hormone was higher in the leaflet group, fitting with the lower vitamin D and the greater risk of osteomalacia in this group.

The numbers who were vitamin D deficient at baseline and at follow-up were similar across groups (Table 3). The mean increase in vitamin D level at follow-up was higher in the leaflet group. Mean vitamin D levels decreased in the DVD group at follow up.

**Table 3:** Vitamin D deficiency and OM

*n* (%)	Group 1 (leaflet)	Group 2 (DVD)
Vitamin D deficient baseline (all / attended both sessions)	15 (94 %) / 11 (92 %)	10 (91 %) / 8 (89 %)
Vitamin D deficient at follow-up	11 (92 %)	9 (100 %)
Vitamin D increase at follow up (mean, 95 % CI)	14.08 (0.54, 27.63)	−15.78 (−34.49, 2.93)
High PTH baseline (all / attended both sessions)	8 (53 %)* / 7 (64 %)*	1 (10 %) / 1 (13 %)
High PTH at follow-up	6 (55 %)	4 (50 %)
PTH decrease at follow-up (mean, 95 %) CI)	0.63 (−2.28, 3.54)	0.24 (−1.90, 2.376)
Biochemical OM at baseline (all / attended both sessions)	8/15 (59 %) / 7/11 (64 %)	1/10 (10 %) / 1/8 (13 %)
Biochemical OM at follow-up	6/11 (55 %)	4/8 (50 %)

The number of people with high PTH levels was much higher in the leaflet group versus the DVD group at baseline and similar to the DVD group at follow-up. The number of people with high PTH levels increased in the DVD group at follow-up.

Biochemical osteomalacia (defined as vitamin D < 75 nmol/L plus PTH > 7.6 pmol/L and calcium <2.6 nmol/L) was more prevalent among the leaflet group at baseline and decreased slightly at follow-up. However, biochemical OM increased at follow-up among the DVD group.

## Conclusions

The key to providing effective health education information is to employ accessible language and multiple formats [22]. We have confirmed that the current AR-UK mind map images are culturally acceptable [17]. This validation is important in the development of a culturally appropriate educational package in order to ensure that the educational method is of real practical value. Our previous work in partnership with ethnic minority community groups supports both the feasibility as well as the benefit of this novel community participatory research approach [16, 17, 23]. The current study is novel in that a layered, culturally acceptable, musculoskeletal, and electronic educational resource delivered in a group community setting has not previously been studied, and there is little evidence of the effectiveness of ‘new media’ approaches among minority ethnic groups [24].

This study tested the feasibility of a community delivered, cluster-randomised trial of a layered pictorial presentation about osteomalacia in an interactive DVD format, and to test if this knowledge translates to better health outcomes and, in particular, good compliance/concordance as evidenced by improved vitamin D and PTH levels. Our results were inconclusive in terms of outcomes and provided evidence for the difficulty in a study of this design. We had hoped that the groups would be similar in most ways; however, it transpired that they were very different in language, both preferred and their use of English and their educational attainment. Osteomalacia knowledge as evidenced by OKQ scores improved more in the leaflet group, whilst biochemical osteomalacia increased at follow-up in the DVD group. However, the DVD group’s OKQ score were substantially higher at baseline and thus had less potential for increase, reflecting the discord between knowledge and behaviour. The increase in osteomalacia among the DVD group remains unexplained, although the period of the study coincided with the end of the winter and may reflect cumulative vitamin D deficiency for this period (the DVD group sessions were done in March/April and the leaflet sessions in January/March).

Although the study was primarily designed as a pilot/feasibility study to test the effectiveness of the interventional DVD, we were interested to note the beneficial effects of using the currently available Arthritis Research UK leaflet on osteomalacia in combination with a concise presentation emphasising key facts. The group receiving this form of education showed a marked improvement in knowledge as shown by a threefold increase in OKQ score. There was also an increase in mean vitamin D levels from 43 to 57 nmol/L. The AR-UK leaflet is a simple, economical and straightforward method of delivering education on osteomalacia, but in this study the leaflet was augmented by a short PowerPoint presentation, and it is unknown if the leaflet alone would have had the same impact.

There were a number of organisational issues that may have affected outcomes. The two centres which agreed to participate insisted on approaching potential participants themselves. They did this by approaching organised groups within the centres: an exercise group for the leaflet group and an English language class for the DVD group. Although we gave instructions as to a recruitment profile, we were unable to ensure that these inclusion criteria had been upheld. Six leaflet participants and two DVD participants reported that they were taking vitamin D at the baseline session. Three participants in the DVD group were from the Middle East and therefore not South Asian.

Each centre provided an additional translator to assist with the completion of forms and questionnaires. As this translator was not research trained, there may have been some bias introduced in the translation of the forms in terms of leading the answers to questions. This may explain the difference in OKQ scores between the groups.

The venues were not ideal for taking blood samples as they were cold and cluttered. Thus, there were insufficient samples taken on four occasions. Participants were paid £10 at the second blood sample and this ensured fairly good compliance.

The leaflet and the DVD sessions both included PowerPoint presentations, but the sessions were led by different tutors (using the same simultaneous Urdu translator). This may have introduced bias in terms of style or methods of presentation. The leaflet presentation was short and concise and the DVD mind map was quite detailed. The leaflet group reported better results despite the fact that the DVD group reviewed the DVD three times as a group between sessions. It is possible that the presentation that accompanied the circulation of the AR-UK osteomalacia leaflet, something that is not routinely provided, has biased the results. It is also possible that the presentation of any information on vitamin D and OM would be beneficial.

The leaflet group sessions were conducted in January and March, and the DVD sessions in March and April. A study such as this must be undertaken between October and April to avoid confounding the results with sun exposure. However, the first session was originally scheduled in December at a third venue but this had to be called off due to snow closing the local schools. That venue was unable to accommodate us on an alternative date.

Community-based settings require a different model for ethical review, nurse time and serum analysis compared with UK National Health Service research studies. Setting up contracts to buy out nurse time and to purchase pathology time was difficult to arrange.

In summary, the difficulties in undertaking this study suggest that even a cluster-randomised design may not be feasible. An individually randomised design had been discarded primarily because of potential cross contamination problems. Recruiting enough groups to control for all confounders seems to be impractical. An alternative way of capturing this population level educational impact is necessary. New research strategies such as the use of trials within cohorts may be appropriate [25]. Despite the challenges that we have identified, our pilot study has shown that the correlation between knowledge and PTH levels is worthy of further study.
